# The Gelation Mechanism of an Apple Polysaccharide at Ambient Temperature as Induced by Ca^2+^

**DOI:** 10.3390/foods15061076

**Published:** 2026-03-19

**Authors:** Shuai Luo, Junhao Qiu, Shuaida Wang, Xi Yang, Haopeng Wang

**Affiliations:** 1 College of Food Engineering and Nutritional Science, Shaanxi Normal University, 620 West Chang’an Avenue, Xi’an 710119, China; luoshuai@snnu.edu.cn (S.L.); sdwang618@snnu.edu.cn (S.W.); 2 State Key Laboratory for Quality and Safety of Agro-Products, Ningbo 315800, China; junhqiu@163.com (J.Q.); yangxi1@nbu.edu.cn (X.Y.); 3 Zhejiang-Malaysia Joint Research Laboratory for Agricultural Product Processing and Nutrition, College of Food Science and Engineering, Ningbo University, Ningbo 315800, China

**Keywords:** apple polysaccharides, gels, gelation, rheology, EPR

## Abstract

In this study, an apple polysaccharide (AP) that exhibited a gelatin-like gelation behavior has been reported, with the gelation mechanism being further revealed. It was found that a suitable amount of Ca^2+^ addition (4.5 mmol·L^−1^) induced the formation of AP gels at 0.5% (*w/v*) polymer concentration in a wide pH range (3.0–8.0) by holding the polysaccharide solution at 4 °C. However, no gel was formed in the absence of Ca^2+^. Meanwhile, all gels melted around 33 °C upon reheating, and the change in pH did not significantly affect the formation and melting processes of the AP gels. Furthermore, ITC and EPR measurements indicated no detectable binding of Ca^2+^ to AP chains. Thus, the gelation mechanism was explained as Ca^2+^-mediated electrostatic screening, whose presence facilitated AP chain–chain association and ultimately triggered network formation. Our results suggested that AP may exhibit high potential as a possible gelatin substitute in food production.

## 1. Introduction

As a typical anionic heteropolysaccharide found in the primary cell walls of higher plants, pectin exhibits a relatively complex and diverse structure, with varied gelation behaviors [1,2]. Commercial pectin extraction primarily relies on citrus peels and apple pomace as raw materials due to their wide availability [3]. The complex structure of pectin depends not only on the plant species of raw materials but also on the extraction techniques and operational parameters, which further influence its applicability in the food industry. For example, high methoxyl pectins can form gels in relatively acidic solutions (e.g., pH ~ 3.5) with high concentrations of sucrose as cosolvent (65%, *w/v* typical) [4]. At low pH, the electrostatic repulsion between pectin molecules is minimized by hydrogen ions, while the water activity is reduced by high-concentration sucrose, thereby promoting crosslinking between pectin chains [5]. On the contrary, the gelation of low methoxyl pectins is primarily induced by divalent cation-bridged crosslinking between carboxylate anions of adjacent pectin chains, a process described by the “egg-box” model [6].

In recent years, efforts have been made to develop new pectins with enhanced functional properties to fulfill the growing needs of the food industry. These pectins generally exhibit gelation behaviors unexpected from commercial apple and citrus pectins [7]. For instance, *Nicandra physalodes* (Linn.) Gaertn. pectin with a high content of glucuronic acid (GalA, 87.8%, *w/w*) and a low degree of methoxylation (DM, 28%) only exhibited strong gelling ability in the presence of Ca(OH)_2_, and neutral calcium salts (e.g., CaCl_2_ and CaSO_4_) were unable to induce the gelation [8]. Creeping fig (*Ficus pumila* Linn.) pectin with 87.73% (*w/w*) GalA and a low DM value (14.6%) could spontaneously form a gel at room temperature without requiring any cosolvent [9]. *Premna microphylla* Turcz pectin with 73.50% (*w/w*) GalA and a low DM value of 13.69% extracted by ammonium oxalate ((NH_4_)_2_C_2_O_4_) exhibited strong gelling ability with modest concentration of Na^+^ addition, but other metal ions, including Ca^2+^ and K^+^, were not effective [10]. These findings suggest that pectins possess versatile gelling properties and show great potential for application in the food industry.

Gelatin is a natural protein primarily derived from the partial hydrolysis of collagen found in bovine and porcine hides, which has been widely applied in numerous food products, such as meat spreads, confectionery, low-fat margarine, and cheese products [11]. The gelation behaviors of gelatin are distinguishably unique, featuring a low gelling temperature and a gel melting point approximating body temperature [12]. Therefore, gelatin-added products usually produce a “thinning” mouthfeel. However, gelatin is unacceptable to vegetarians and groups with special religious beliefs [13]. Therefore, it is promising to search for gelatin substitutes from natural polysaccharides [14]. To achieve this target, at least two criteria should be met: (1) the polysaccharide should melt at a temperature comparable to body temperature; (2) the gel should be transparent and elastic once the gel is formed [13]. However, it is extremely difficult for most gelling polysaccharides to meet the two criteria simultaneously due to the low possibility of balancing the gel strength and gel-melting process [15]. For example, for the most widely used gelling polysaccharides, such as gellan gum, carrageenan, and agarose, enhancing gel strength (by increasing polymer concentration, adding metal cations, and so on) often inevitably increases the gel’s thermal stability, making it almost impossible to achieve the relatively low melting temperature [13].

In our recent work, we discovered a commercial apple polysaccharide that was industrially produced by employing metal ion precipitation technology but exhibited gelation properties very similar to those of most gelatins [16]. Structurally, this polysaccharide consists primarily of neutral sugars and a minor fraction of acidic sugars (1.61% glucuronic acid) with a DM of 12.5%. Due to the low amount of acidic sugar, it is not proper to term the polysaccharide as “pectin”. Thus, we term it as “apple polysaccharide (AP)” to avoid causing misunderstanding. Moreover, it was also found that AP exhibited strong gelling capacity by cooling the hot polymer solution at a low temperature (4 °C) in the presence of a suitable concentration of Ca^2+^ (0.05%, *w/v*). However, the systematic effect of pH variation on the gelation process and the underlying gelation mechanism of the AP-Ca^2+^ system is still unclear.

The present work aimed to further investigate the gelation behavior of the AP-Ca^2+^ system by rheological testing with a wide pH range (3.0–8.0) and revealed the gelation mechanism of AP gels induced by Ca^2+^ via the results of isothermal titration calorimetry and electron paramagnetic resonance measurements. Our research is expected to provide a deeper understanding of the gelation behavior of apple polysaccharides for their industrial application.

## 2. Materials and Methods

### 2.1. Materials

Pectin (CAS number: 9000-69-5) from apple sources extracted via the metal ion precipitation method was obtained from Shanghai Yuanye Bio-Technology Co., Ltd. (Shanghai, China), but the residual metal ions were not pre-removed. To better research the gelation behavior and mechanism of AP, purification of the product was carried out according to a previously reported method [16]. Briefly, 1.0% (*w*/*v*) crude AP solution was mixed with three volumes of 95% (*v/v*) ethanol. The precipitate was rehydrated in deionized water, dialyzed using a 3 kDa cut-off membrane (MD 45 mm), and finally freeze-dried. The purified AP has a molecular weight (Mw) of 361.7 kDa with a monosaccharide composition including neutral sugars (glucose, mannose, galactose, and rhamnose) and a small amount of glucuronic acid (1.61%), with final yields of 20–30%. After purification, the contents of Na^+^, K^+^, and Ca^2+^ were reduced to 0.07%, 0.05%, and 0.97% (*w/w*), respectively, from the initial levels of 0.31%, 16.57%, and 2.10% (*w/w*) [16]. Other analytical grade chemical regents, including CaCl_2_, NaOH, HCl, MnCl_2_·4H_2_O, and CuSO_4_·5H_2_O were provided by Tianjin Tianli Chemical Reagents Co., Ltd. (Tianjin, China). KBr (spectroscopical grade) was purchased from Sigma Aldrich (St. Louis, MO, USA). Deionized water was employed consistently in all experiments unless stated otherwise.

### 2.2. Gel Preparation

To reveal the effect of pH variation on the AP gelation process, 1.5 g of AP was dissolved in 300 mL of deionized water to prepare a 0.5% (*w/v*) AP solution, and the solution was continuously heated and stirred magnetically at 80 °C for 2 h to ensure full dissolution of the pectin. Then, the AP solution was divided into six equal aliquots and adjusted to pH values of 3.0, 4.0, 5.0, 6.0, 7.0, and 8.0 using 0.5 mol·L^−1^ NaOH and 0.5 mol·L^−1^ HCl solutions. Afterward, AP solutions were separately mixed with 25 mg of CaCl_2_ at 25 °C for 12 h. Then, another group of identically processed solutions was held at 4 °C for 12 h to achieve adequate gelation. Considering that the addition amount of HCl and NaOH solution is negligible, the concentration of AP (0.5%, *w/v*) and CaCl_2_ (0.05%, *w/v*, equivalent to 4.5 mmol·L^−1^ Ca^2+^) in the final gels is considered unchanged.

### 2.3. Scanning Electron Microscope Observations

The microstructure of AP gels under different pH values was visualized using a scanning electron microscope (SEM, Quanta 200, FEI Company, Hillsboro, OR, USA). AP gels prepared as described in Section 2.2 were immersed in liquid nitrogen for rapid freezing to fix the gel microstructure, followed by dehydration via a vacuum freeze dryer (FD-1A-50, Beijing Boyikang Experimental Instrument Co., Ltd., Beijing, China) at −50 °C for 48 h. After the lyophilized gels were fractured and sprayed with a thin layer of gold powder, the morphology of the fracture section was visualized [17].

### 2.4. Rheological Measurement

The gelation and gel-melting behaviors of AP at pH 3.0–8.0 were monitored using a rheometer (MCR 302, Anton Paar GmbH, Graz, Austria) equipped with a 50 mm diameter rough-surfaced parallel plate. After the AP solution was mixed with Ca^2+^, approximately 2.0 mL of the mixture solution was added onto the parallel plate preheated to 50 °C. Then, the gap of the plate (1 mm) was sealed with suitable low-density silicone oil to avoid water evaporation. After holding at 50 °C for 10 min to achieve equilibrium, the sample was cooled to 4 °C at a slow rate (1 °C/min), followed by holding at 4 °C for 7200 s (2 h), after which the sample was heated to 60 °C at a slow rate (1 °C/min). All tests were consistently conducted at a frequency of 1 Hz and a strain of 1.0%, thus remaining in the linear viscoelastic range [18,19].

### 2.5. Thermogravimetric Analysis

Thermogravimetry (TG) and derivative thermogravimetry (DTG) measurements of the lyophilized AP gels were conducted by a thermal analyzer (Q600SDT, TA Instruments, New Castle, DE, USA). Briefly, 10.0 mg of the dried AP gels was put on an aluminum crucible at room temperature and heated to 600 °C at a rate of 10 °C/min, using another empty aluminum crucible as the reference and pure nitrogen as the purge gas.

### 2.6. Fourier Transform Infrared Spectroscopy Analysis

For Fourier transform infrared spectroscopy (FTIR) analysis, approximately 5 mg of the dried AP gels was mixed with KBr at a 1:100 ratio, followed by grinding the mixture into powder, which was subsequently pressed into a semi-transparent pellet. Then, the pellet was scanned using a Fourier transform infrared spectrometer (Tensor 27, Bruker Optics GmbH, Ettlingen, Germany) over the wavenumber range of 4000–400 cm^−1^, with 32 scans and at a resolution of 4 cm^−1^.

### 2.7. X-Ray Diffraction (XRD) Analysis

Moreover, in order to analyze the crystal structure and molecular arrangement characteristics of Ca^2+^ induced AP gels under pH 3.0–8.0, an X-ray Diffractometer (D8 Advance, Bruker AXS GmbH, Karlsruhe, Germany) fitted with CuKα radiation source and a nickel filter was utilized to conduct X-ray diffraction measurement from 5° to 60° at 0.02° of step size.

### 2.8. Atomic Force Microscope (AFM) Observations

The molecular morphology of AP was observed using an atomic force microscope (Dimension ICON, Bruker Nano Surfaces & Metrology GmbH, Karlsruhe, Germany) at a wide pH value with or without the addition of Ca^2+^ [20,21]. To begin with, an extremely diluted AP solution (0.001%, *w/v*) was prepared, and the pH was sequentially adjusted to 3.0, 4.0, 5.0, 6.0, 7.0, and 8.0. After adding a suitable amount of CaCl_2_ in the AP solutions to achieve 4.5 mmol·L^−1^ Ca^2+^ concentration (as consistent with the identical Ca^2+^ environment in AP gels), 10 μL of the AP solution was dried at room temperature for 24 h on a fresh mica sheet. In the meantime, the pure AP solutions without added Ca^2+^ served as control. Afterward, AFM was performed to image the sample in tapping mode [22].

### 2.9. Isothermal Titration Calorimetry (ITC) Analysis

An isothermal titration calorimeter (Nano ITC, TA Instruments, New Castle, DE, USA) was utilized to analyze the enthalpy change in the interaction between AP and metal ions. Briefly, 350 μL of 0.1% (*w/v*) AP solution was added into the ITC cell mixed with 20 consecutive dropwise injections of 45.0 mmol·L^−1^ Ca^2+^ solution pre-added in ITC Titration Syringe at 300 rpm [23]. Each injection volume was 2.5 μL, with a duration of 2 s and an interval of 300 s to ensure complete reaction. Meanwhile, 350 μL of deionized water, subjected to the same 45.0 mmol·L^−1^ Ca^2+^ solution injection, served as the blank. Corrected heat data were derived by integrating the peak areas, with subsequent subtraction of the dilution enthalpy obtained from the blank test [24].

### 2.10. Electron Paramagnetic Resonance (EPR) Analysis

EPR is also known as electron spin resonance (ESR), capable of detecting the paramagnetic signal of transition metal ions with unpaired electrons such as Mn^2+^ and Cu^2+^ [25]. However, Ca^2+^ cannot be detected by EPR spectroscopy due to the lack of a paramagnetic signal. Despite this, given that Mn^2+^, Ca^2+^, and Cu^2+^ all belong to group II cations, EPR measurement on Mn^2+^ and Cu^2+^ in AP solution is still valuable to infer information about the interaction of Ca^2+^ with the carboxylic groups on AP chains.

Prior to EPR analysis, it is essential to investigate the impact of Mn^2+^ and Cu^2+^ on AP gelation. Briefly, 0.5% AP solutions containing 0, 1.8, 4.5, 9.0, 18.0, and 45.0 mmol·L^−1^ Mn^2+^ and Cu^2+^ were separately prepared at room temperature, followed by holding at 4 °C for 12 h to achieve equilibrium. Then, the occurrence of gelation was assessed by the tube inversion method.

For EPR analyses, the spectra of 0.05 and 4.5 mmol·L^−1^ Mn^2+^ (or Cu^2+^) in 0.5% AP solution (*w*/*v*) were procured by employing an X-band EPR Spectrometer (Bruker E500, Bruker Magnetics GmbH, Karlsruhe, Germany) at both 25 °C and 4 °C, respectively. Relatively low (0.05 mM) and high (4.5 mM) Mn^2+^ (or Cu^2+^) concentrations were considered because a low concentration of Mn^2+^ ion is more likely to bind pectin molecules, and a relatively high concentration of Mn^2+^ induced gel formation of AP, as in the case of the addition of 4.5 mmol·L^−1^ Ca^2+^. Meanwhile, the pure 0.05 and 4.5 mmol·L^−1^ Mn^2+^ (or Cu^2+^) solutions were used as controls. The operational parameters included microwave frequency 9.43 GHz, sweep time 92.16 s, receiver gain 30 dB, field modulation frequency 100.0 kHz, field modulation amplitude 3.0 G, central field 3480.0 G, sweep width 1500.0 G, g-factor 2.0, and power 20.0 mW.

### 2.11. Zeta-Potential Measurement

The Zeta-potential of AP solutions with different CaCl_2_ concentrations was measured by a Zeta-Potential Analyzer (Malvern Zetasizer Nano ZS90, Malvern Panalytical Ltd., Malvern, UK). Briefly, 50 mL of dilute AP solutions (0.1%, *w/v*) containing 0, 1.8, 4.5, 9.0, 18.0, and 45.0 mmol·L^−1^ CaCl_2_ were prepared by mixing 10 mL of the stock AP solution (0.5%, *w/v*) and 40 mL of 0, 2.25, 5.625, 11.25, 22.50, and 56.25 mmol·L^−1^ CaCl_2_ solutions. Afterward, 1 mL of the prepared solutions was used for Zeta-potential measurements at 25 °C.

### 2.12. Statistical Analysis

All measurements were repeated at least three times, with results expressed as the mean ± standard deviation. All figures were plotted using Origin software (Origin 2024 SR1, Version 10.100173, OriginLab Corporation, Northampton, MA, USA).

## 3. Results

### 3.1. Gel Appearance and Microstructures

**Figure 1:**
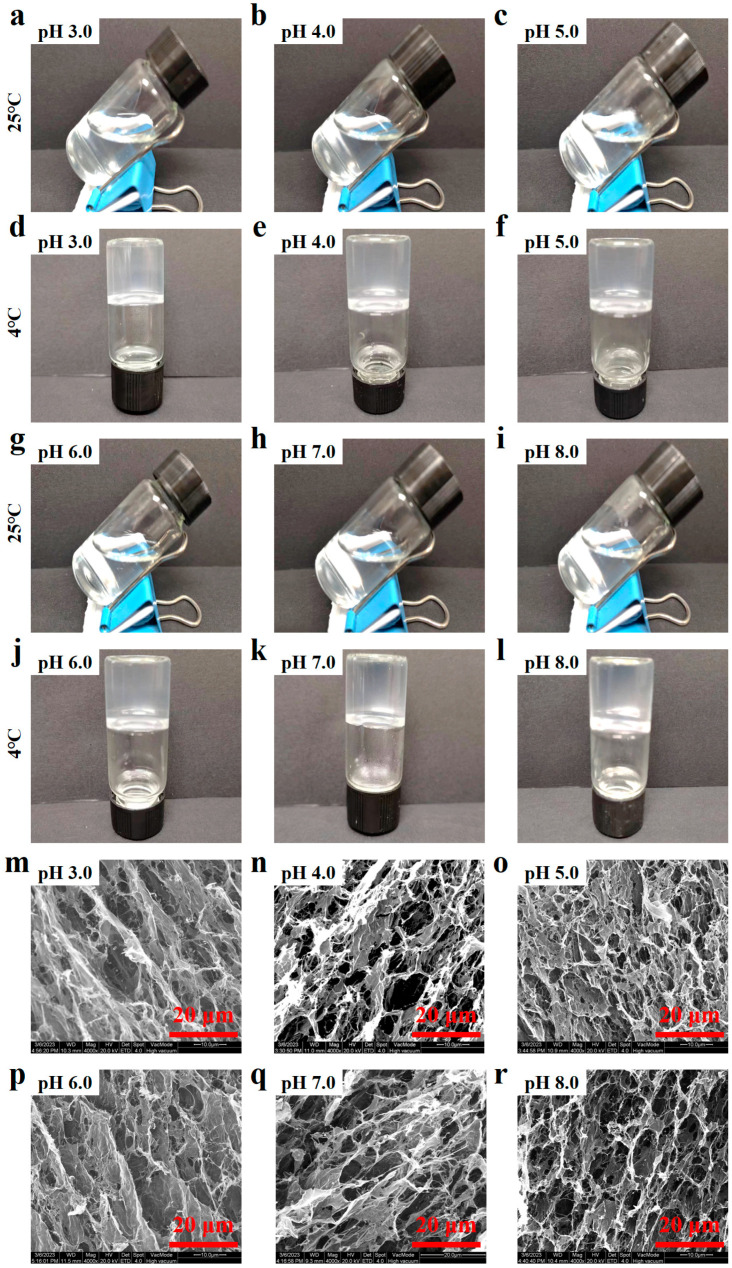
Appearances (**a**–**l**) of AP solutions at 25 °C and AP gels at 4 °C with 4.5 mmol·L^−1^ CaCl_2_ addition at pH 3.0–8.0 and SEM images (**m**–**r**) of lyophilized AP gels with 4.5 mmol·L^−1^ CaCl_2_ addition at pH 3.0–8.0.

At 25 °C, no gel formation was observed for the 0.5% AP solution with coexisting 4.5 mmol·L^−1^ Ca^2+^ at pH 3.0–8.0 (Figure 1). Upon cooling to 4 °C, however, AP solutions formed semi-transparent gels at all pH values, indicating that AP-Ca^2+^ systems can only form gel at low temperature. SEM images at 4000 magnifications showed that, at pH 3.0, the AP gel exhibited a discernible network structure with numerous small fiber-like objects. As the pH value increased to 5.0, the network structure became increasingly discernible; with a continuous increase in pH, the gel network became more uniform, especially at pH 8.0. However, changes in the microstructure are still considered to show no significant differences, unlike the reported transition from disordered to ordered networks in LMP-Ca^2+^ gels under similar pH conditions [19], indicating less pH dependence of AP gels than the LMP-Ca^2+^ gel.

### 3.2. Rheological Properties

**Figure 2:**
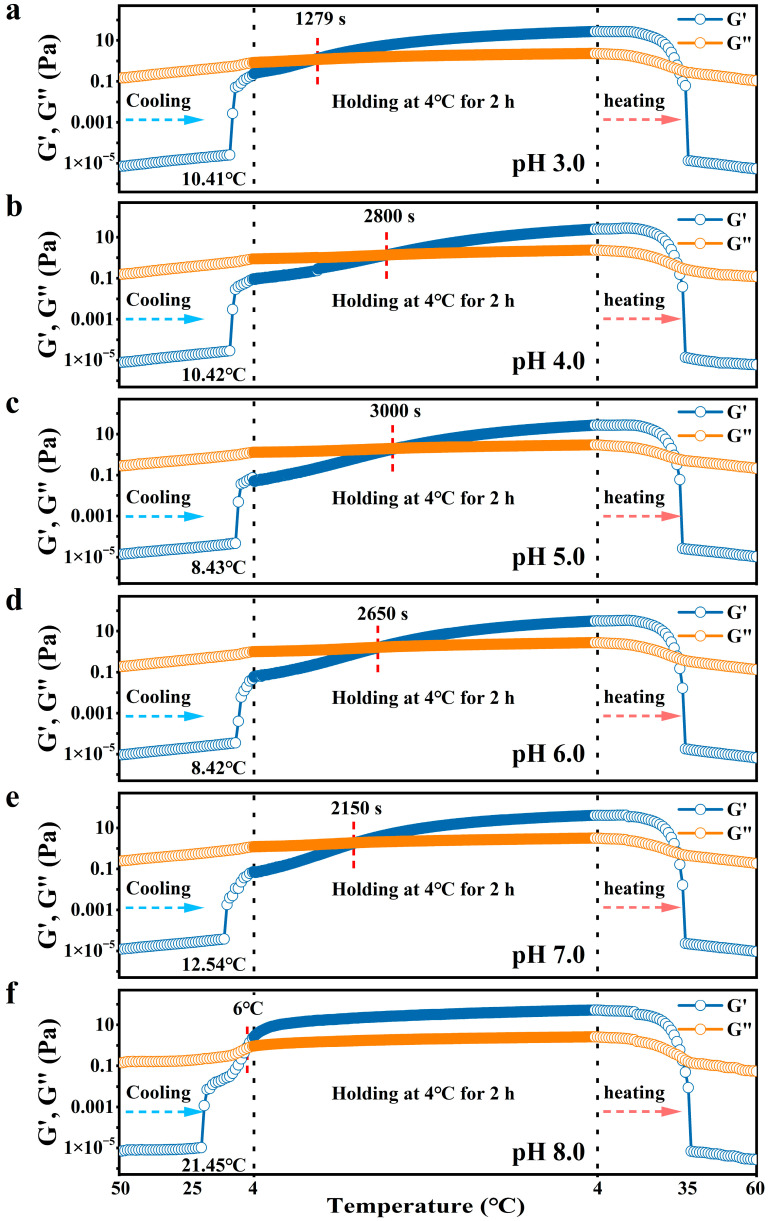
Gelation process and gel-melting process of AP gels with 4.5 mmol·L^−1^ CaCl_2_ addition at (**a**) pH 3.0; (**b**) pH 4.0; (**c**) pH 5.0; (**d**) pH 6.0; (**e**) pH 7.0; (**f**) pH 8.0.

Variations in the storage modulus G′ and loss modulus G″ of the AP-Ca^2+^ systems were monitored during cooling from 50 to 4 °C, isothermal holding at 4 °C, and subsequent reheating to 60 °C. As presented in Figure 2, the G′ of the AP solution at pH 3.0 showed a steep increase when cooling to around 11 °C, followed by a continuous and slow elevation in G′ upon further cooling the solution to 4 °C, suggesting the emergence of significant intermolecular aggregation. At this temperature range, however, G′ was found to be always lower in value than G″, indicating that the intermolecular aggregation was not strong enough to induce gelation. When holding the pectin solution at 4 °C, both G′ and G″ values exhibited a continuous increase, with G′ rising more rapidly than G″, exhibiting a single G′-G″ crossover (G′=G″), a point conventionally approximated as the gelation temperature (T_gel_) [26].

Since the gelation point of all AP solutions only appeared during the holding stage, the T_gel_ actually reflected the gelation time at 4 °C, which was recorded as the time interval between the onset of the holding stage and the time point corresponding to the occurrence of G′=G″. For the AP solution with pH 3.0, T_gel_ was found to be 1279 s. After reaching T_gel_, the upward trend of G′ remained steeper than that of G″, indicating an ongoing evolution of the gel network. Upon the completion of the isothermal holding period, the value of G′ increased to 27.15 Pa, which reflected the gel strength. On reheating, both G′ and G″ were observed to begin decreasing rapidly around 20 °C, but the decrease in G′ was much steeper than that of G″, thus showing another G′=G″ crossover point at 32 °C, conventionally designated as the gel melting temperature (T_melt_).

For the AP-Ca^2+^ system at pH 5.0, the temperature corresponding to the initiation of the significant G′ enhancement slightly shifted to 9 °C during cooling. During the holding stage at 4 °C, T_gel_ was observed at 3000 s, and the G′ value reached a maximum of 27 Pa. On reheating, T_melt_ was also observed around 32 °C. This result suggests that increasing the pH value from 3.0 to 5.0 slightly delayed the gelation of the AP-Ca^2+^ system but did not discernibly affect the gel strength and T_melt_.

As the solution’s pH rose further to 8.0, however, the temperature corresponding to the initiation of the significant G′ enhancement gradually increased to 21 °C during cooling, and the G′=G″ crossover point appeared at 6 °C. Moreover, upon holding at 4 °C for 2 h, the value of G′ slightly increased to 52.14 Pa, indicating that the gelation of AP was slightly promoted at higher pH values. Interestingly, for the AP-Ca^2+^ system at pH 8.0, T_melt_ was observed around 33 °C. Changing pH essentially affects the charge density and chain conformation of AP molecules, which further affects the association of polymer chains [19,27]. Due to the low charge density, it is considered that the variation in pH only slightly affected the intermolecular aggregation and gelation process of AP, implying that AP possessed a good pH resistance.

### 3.3. TG/DTG Analysis

**Figure 3:**
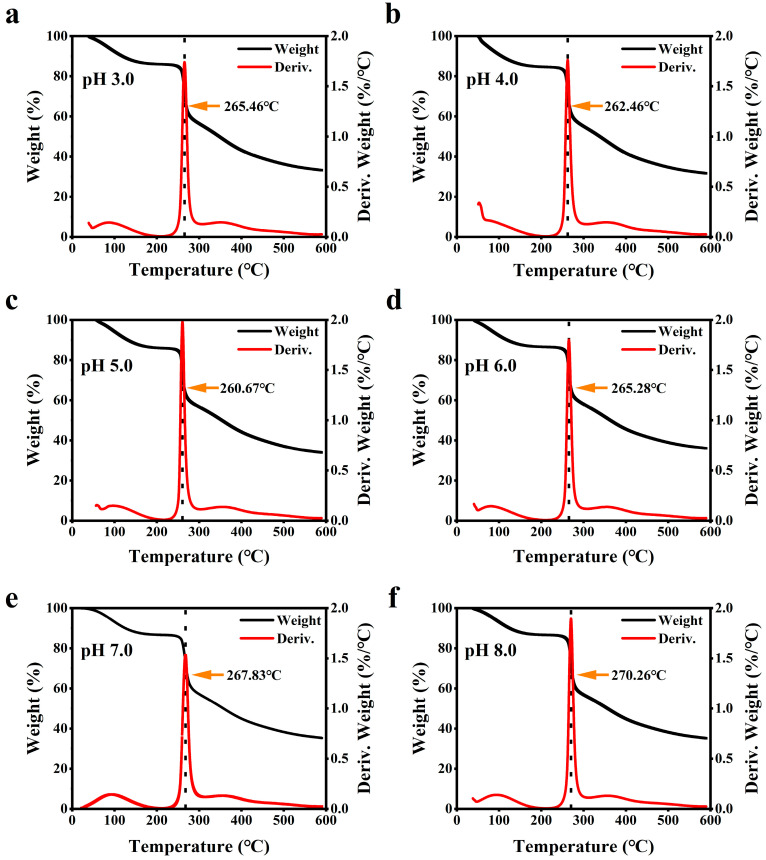
TG/DTG analyses of lyophilized AP gels with 4.5 mmol·L^−1^ CaCl_2_ addition at (**a**) pH 3.0; (**b**) pH 4.0; (**c**) pH 5.0; (**d**) pH 6.0; (**e**) pH 7.0; (**f**) pH 8.0. The dashed line in each subfigure marks the temperatures corresponding to the maximum weight loss rate.

To examine the thermal degradation behaviors of polymers and gels, thermogravimetric techniques (TG/DTG) are typically employed [28]. Within the pH range of 3.0–8.0, the weight of all samples exhibited two distinct segments of significant reduction (Figure 3). The first weight loss stage occurred below 200 °C, resulting from the vaporization of the residual moisture in dried AP gels [29]. Another weight loss stage occurred at 200–300 °C, which was attributed to thermal decomposition of AP molecules [30]. Meanwhile, the temperature corresponding to the maximum thermal degradation of the sample decreased slightly from 265 °C (pH 3.0) to 261 °C (pH 5.0) and then showed a slight increase to 270 °C (pH 5.0). This result indicates that a more acidic environment, below pH 5.0, induced a slight increase in the crosslink density of AP gel, but a pH increase over pH 5.0 resulted in enhanced crosslink density, consistent with the result of rheological measurement.

### 3.4. FTIR and XRD Analyses

**Figure 4:**
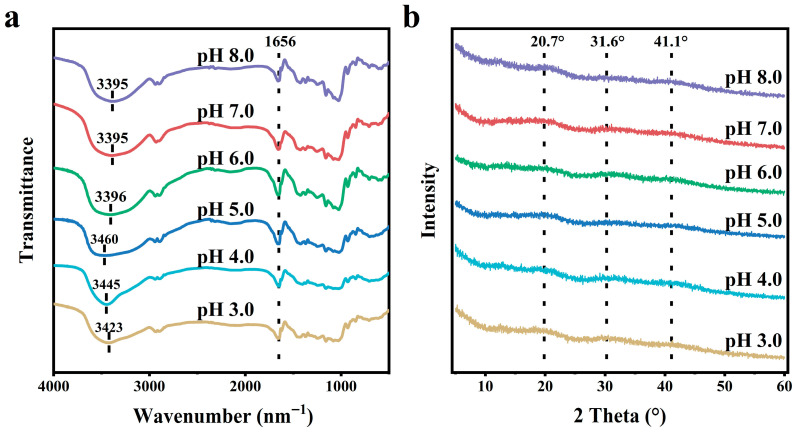
FTIR spectra (**a**) and XRD patterns (**b**) of lyophilized AP gels with 4.5 mmol·L^−1^ CaCl_2_ addition at pH 3.0–8.0.

Figure 4a demonstrated that a prominent absorption peak around 3400 cm^−1^ was observed for all dried AP gels, which arises from the broad O-H stretching band of hydroxyl groups (-OH) within the polymer. Additionally, another prominent band located at 1656 cm^−1^ was also observed, corresponding to C=O stretching mode of nonesterified carboxyl groups (-COOH) [31]. As the pH increased (<pH 5.0), the absorption band of O-H stretching vibrations exhibited a systematic redshift from 3423 cm^−1^ (pH 3.0) to 3460 cm^−1^ (pH 5.0), indicating that intermolecular hydrogen bonding in AP gel was gradually weakened [10]. In contrast, the observed redshift to lower wavenumbers of 3396 cm^−1^ after the pH value increased to 6.0 indicated the re-establishment of an intensified intermolecular hydrogen bonding. As the pH value further increased to 8.0, the shift in the O-H stretching vibration band was very slight, implying the negligible intensification of intermolecular hydrogen bond formation and disruption in AP gel.

To characterize the crystalline structure of dried AP gels prepared at different pH values, XRD patterns are displayed in Figure 4b. As observed, all gels exhibited three broad peaks at 2θ≈20.7°, 31.6°, and 41.1°, respectively. The lack of sharp diffraction peaks confirmed an amorphous/semi-crystalline structure in AP gels [32]. Moreover, the sharp characteristic peaks of CaCl_2_ were absent in all samples, probably because the addition amount of CaCl_2_ is too low to allow the formation of CaCl_2_ crystals [19].

### 3.5. Molecular Morphology

**Figure 5:**
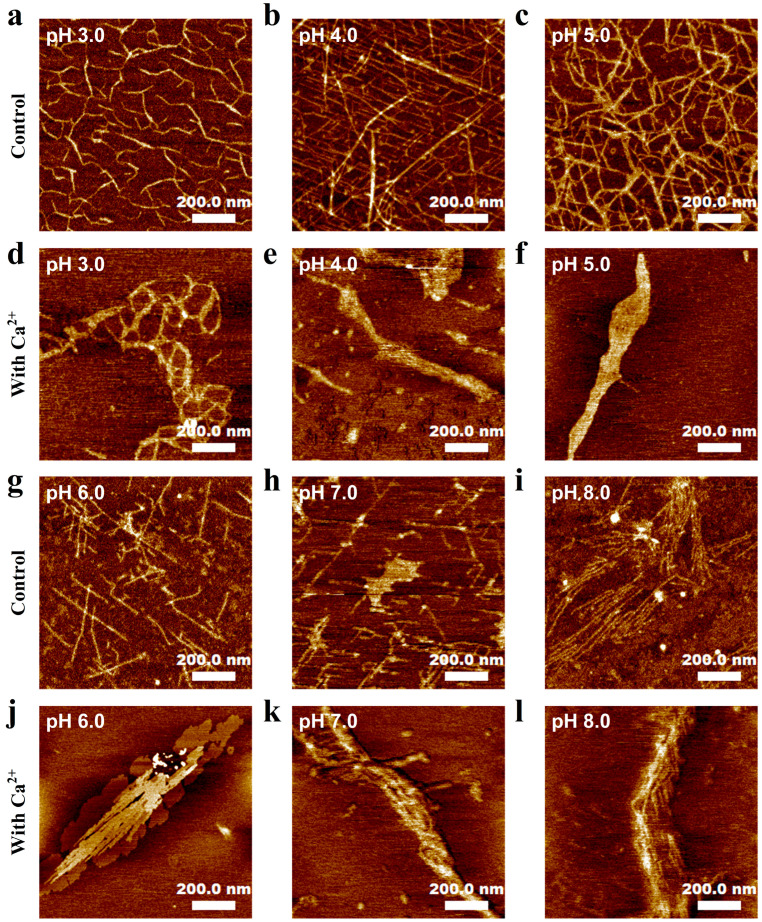
AFM images of AP molecules at (**a**–**c**) pH 3.0–5.0 without CaCl_2_ addition; (**d**–**f**) pH 3.0–5.0 with 4.5 mmol·L^−1^ CaCl_2_ addition; (**g**–**i**) pH 6.0–8.0 without CaCl_2_ addition; (**j**–**l**) pH 6.0–8.0 with 4.5 mmol·L^−1^ CaCl_2_ addition.

For the pure AP solutions (control), the molecules existed as untangled and fiber-like multi-branch chains at pH 3.0 (Figure 5). Upon increasing the pH to 5.0, AP molecules exhibited an increasing extent of aggregation, showing an entangled network structure. When the pH value further increased to 8.0, the entangled network gradually disappeared, and AP molecules seemed to become more rigid. In the presence of Ca^2+^, AP molecules displayed very pronounced intermolecular aggregation at all pH values. Especially, as pH increased, the level of intermolecular aggregation showed a slight upward trend, indicating that Ca^2+^ supplementation greatly boosted the intermolecular aggregation of AP compared to Ca^2+^-free controls.

### 3.6. ITC Analysis

**Figure 6:**
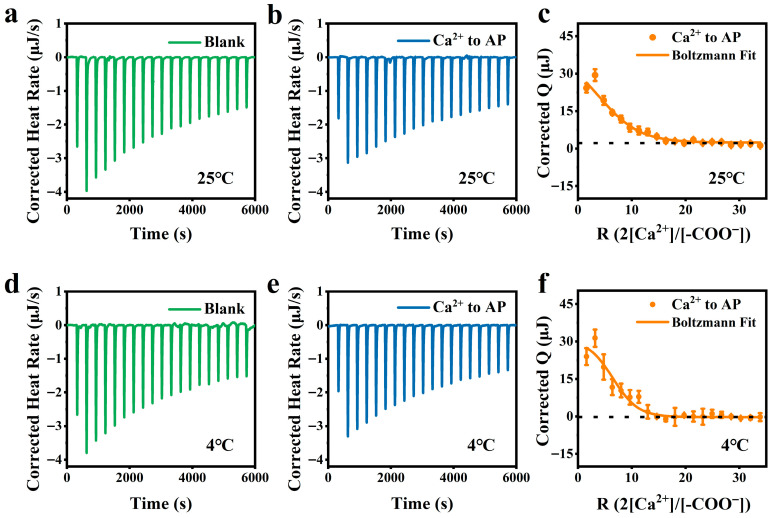
Blank ITC thermograms obtained by injecting 45.0 mmol·L^−1^ CaCl_2_ solution into deionized water, ITC thermograms of injecting 45.0 mmol·L^−1^ CaCl_2_ solution into 0.41 mmol·L^−1^ AP solution, and Corrected Q curves of Ca^2+^ binding to AP (Boltzmann fit) at 25 °C (**a**–**c**) and 4 °C (**d**–**f**). The dashed line in each subfigure denotes the zero reference baseline for the corrected Q value.

ITC is a powerful tool for studying salt–polysaccharide interactions through measuring the endothermic or exothermic heat flow during mixing of the polysaccharide solution with consecutive dropwise injections of the salt solution [33]. As shown in Figure 6, the injection peak became sharper at the second injection of Ca^2+^ solution, and then it gradually decreased. At 25 °C, the ITC thermogram of the titration of Ca^2+^-AP solution exhibited strong similarity to that of Ca^2+^ titration into water (blank) (Figure 6a,b). This phenomenon indicated that, when Ca^2+^ was titrated into AP solution, the main heat changes still came from the dilution heat of Ca^2+^ in water, and the effect of interaction between Ca^2+^ and AP molecules was almost negligible. Following background subtraction of the heat effect of the metal ion dilution experiment, the calibrated ITC data demonstrated that the Ca^2+^-AP interactions caused mild absorption of the heat during the initial 10 titration steps (Figure 6c), unlike the reported multi-step exothermic reaction attributed to the binding of Ca^2+^ to LMP [23]. Therefore, the observed ITC pattern of Ca^2+^ titration into AP is not considered as the result of ion crosslinks. A similar ITC pattern was also observed for the AP solution with dropwise injections of Ca^2+^ solution at 4 °C (Figure 6d–f), indicating that the interaction of AP molecules with Ca^2+^ at 4 °C was also a pure electrostatic interaction.

### 3.7. EPR Analysis

**Figure 7:**
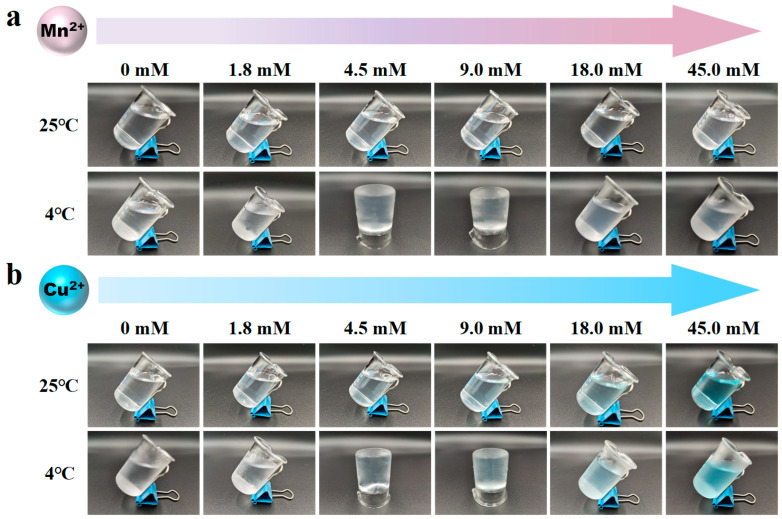
Appearance of AP solutions at 25 °C and AP gels at 4 °C with different Mn^2+^ (**a**) and Cu^2+^ (**b**) concentrations.

We further performed EPR measurements to confirm the interaction between Ca^2+^ and AP molecules. For EPR spectroscopy, the paramagnetic signal of a transition metal ion such as Mn^2+^ or Cu^2+^ will be annihilated if the metal ion binds to anionic polysaccharide chains [34]. As Ca^2+^ is not a paramagnetic metal ion and cannot be detected by EPR spectroscopy, the binding affinity of divalent cations to the carboxylic groups follows the order of Mn^2+^ < Ca^2+^ < Cu^2+^. We used Mn^2+^ and Cu^2+^ in place of Ca^2+^ to reveal the interaction mechanism of divalent metal ions with AP molecules [25]. Before measurement, it was important to consider whether the incorporation of Mn^2+^ and Cu^2+^ also showed a similar ability to induce AP gelation as observed for AP-Ca^2+^ systems. For this reason, a wide concentration range of Mn^2+^ and Cu^2+^ was considered. As shown in Figure 7, at a 4.5–18.0 mmol·L^−1^ Mn^2+^ and Cu^2+^ concentration range, AP could not form a gel at 25 °C. Upon cooling to 4 °C, gel formation was observed. Further addition of Mn^2+^ and Cu^2+^, however, gradually weakened and ultimately inhibited the gelation, mirroring the concentration-dependent effect of Mn^2+^ and Cu^2+^ on AP gelation, a result highly consistent with the reported effect of Ca^2+^ on AP gelation [16]. Therefore, it is considered that Cu^2+^, Mn^2+^, and Ca^2+^ should exert a very similar effect on AP gelation, implying that the interaction of Ca^2+^ with AP molecules can be reasonably inferred from the result of EPR measurement on Cu^2+^ and Mn^2+^.

**Figure 8:**
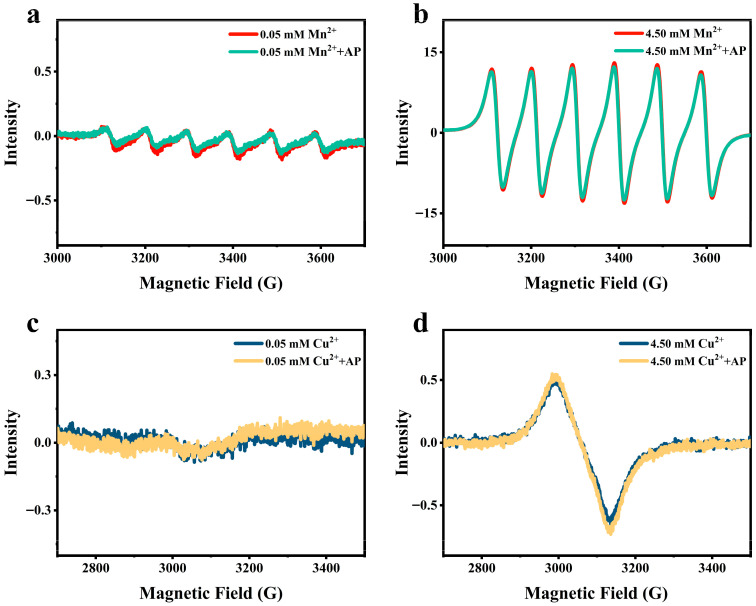
EPR spectra of different concentrations of Mn^2+^ (**a**,**b**) or Cu^2+^ (**c**,**d**) solutions at 4 °C with or without 0.5% AP addition.

As shown in Figure 8a,b, both 0.05 and 4.5 mmol·L^−1^ Mn^2+^ solutions presented a typical six-line spectrum, arising from the electron–nuclear hyperfine coupling between the unpaired electron spin and the I = 5/2 nuclear spin of Mn^2+^ [25]. In the AP solution, the characteristic six-line spectrum of both 0.05 and 4.5 mmol·L^−1^ Mn^2+^ almost remained unchanged, suggesting that Mn^2+^ actually did not bind to the carboxylic groups on AP molecules. The EPR spectrum of 0.05 and 4.5 mmol·L^−1^ Cu^2+^ solutions exhibited a typical two-line spectrum. The first line possessed 200 G of field width with a central field at 2986 G (g-factor = 2.256). The second line possessed only 5 G of field width with a central field at 3360 G (g-factor = 2.005) (Figure 8c,d). The two lines corresponded to the effect of spin-orbit coupling between the unpaired electron spin and Cu^2+^ nuclear spin at two inequivalent sites in copper sulfate pentahydrate [35,36]. In 0.5% AP solution, the EPR spectrum of both 0.05 and 4.5 mmol·L^−1^ of Cu^2+^ also remained unchanged, indicating that Cu^2+^ did not bind to the carboxylic groups on AP molecules. Considering that the binding affinity of divalent cations to the carboxylic groups follows the order of Mn^2+^ < Ca^2+^ < Cu^2+^ [37,38], it is reasonable to assume that Ca^2+^ also did not bind to the carboxylic groups on AP chains.

## 4. Discussion

As reported in our earlier research, we characterized the gelation capability of AP in solutions with coexisting Ca^2+^. It was found that AP could form a gel with moderate concentration of Ca^2+^ addition (0.05–0.1%, *w/v*) coupled with cooling to 4 °C. At higher concentrations of Ca^2+^, however, the gelation was abolished [16]. We explained the gelation behavior as the delicate balance between the formation of intermolecular hydrogen bonding and the interaction of Ca^2+^ with AP chains. However, the mechanism by which Ca^2+^ affects AP gelation remains undefined. Herein, we conducted the present study to further reveal how pH variation modulates the gelation behavior of the AP-Ca^2+^ system and elucidated the role of Ca^2+^ in the gelation process. It was found that, in the absence of Ca^2+^, changing the pH value alone failed to trigger the gelation of the polysaccharide system at both 25 and 4 °C (Figure S1). In contrast, when 4.5 mmol·L^−1^ Ca^2+^ was presented, all AP solutions formed gel across a broad pH range (3.0–8.0) at 4 °C (Figure 1).

The results of rheological measurement suggest that, with pH increasing from 3.0 to 8.0, the gelation time at 4 °C exhibited a decrease followed by an increase with a critical turning point at pH 5.0. This observation indicated that low pH values (pH < 5.0) slightly promoted AP gelation by decreasing the dissociation extent of the carboxylic groups on AP chains, which enhanced the chain–chain association of AP. At high pH values (pH > 5.0), although the dissociation extent of the carboxylic groups increased, which is supposed to increase the electrostatic repulsion between AP chains, the presence of Ca^2+^ can possibly bind to AP chains or produce a greater extent of electrostatic shielding effect due to its bigger cation radius to promote AP gelation [39]. Despite this, however, the overall effect of changes in pH on AP gelation is slight, and we observed that the T_gel_ and T_melt_ of AP were similar at the pH range studied (Figure 2). We ascribed the reason to the low charge density of AP, which is believed to impart AP low sensitivity to pH variation [16].

Moreover, we noticed that, in the presence of Ca^2+^, AP showed intermolecular aggregation at all pH values (Figure 5). Thus, we assumed that Ca^2+^ exerted its effect by two possible mechanisms: (1) electrostatic shielding, which mitigated the repulsion between AP chains, thereby facilitating chain aggregation via intermolecular hydrogen bonding; and (2) direct binding to AP chains to form intermolecular ionic crosslinks as usually observed for other LMP-Ca^2+^ systems. By determining the ζ-potential of AP in Ca^2+^-supplemented systems, we found that a concentration increase in Ca^2+^ from 0 to 4.5 mmol·L^−1^ induced a rapid reduction in the ζ-potential of AP from −24.4 mV to −9.72 mV; as the Ca^2+^ concentration continuously increased to 90.0 mM, the ζ-potential approached zero, indicating that a small amount of Ca^2+^ addition significantly reduced the charge density of AP (Figure S2). This phenomenon further corroborated that an excessively high Ca^2+^ concentration induced charge annihilation of AP chains, minimized intermolecular repulsive forces between adjacent chains, triggered excessive molecular aggregation, and thus abolished gelation. Moreover, by performing ITC and EPR measurements, we demonstrated that there was no detected binding of Ca^2+^ to AP chains (Figure 6 and Figure 8). Therefore, the interaction of Ca^2+^ with AP chains is considered as a pure electrostatic interaction, which also explains the relatively low T_melt_ of all AP gels upon heating.

Moreover, it is worth mentioning that, in our recent publication, the effect of monovalent cations was also considered. It was found that AP showed strong gelling capacity with increasing concentrations of K^+^, Cr^+^, and Rb^+^, but with the addition of Na^+^, AP gelation was absent, even at elevated ionic contents [40]. In our present work, it seems that Mn^2+^, Ca^2+^, and Cu^2+^ exhibited similar gel-inducing capacity (Figure 7), with only medium concentrations of divalent cations showing efficacy to induce gelation. This observation suggested that both ionic radius and valency can affect the electrostatic shielding effect of metal cations on AP chains, according to Manning theory [41]. This mechanism deserves further investigation and should be the essential reason explaining the gelation behavior of AP. In spite of this, from the viewpoint of food application, the good pH resistance and low gel-melting temperature of AP-Ca^2+^ systems may be appreciated when seeking a substitute for gelatins from natural polysaccharides.

## 5. Conclusions

This research investigated the gelation behavior and mechanism of AP in solution with coexisting Ca^2+^ (4.5 mM) across a broad pH range (3.0–8.0). It was found that AP could form a gel at 4 °C. On reheating, the gel melted around 32 °C; the gelation process possessed good pH resistance. Based on the results of ITC and EPR measurements, the pure electrostatic interaction between Ca^2+^ and AP chains was considered as the predominant gelation mechanism. Upon cooling AP solutions at sufficiently low temperatures (e.g., 4 °C), Ca^2+^ is believed to promote AP chain–chain aggregation by decreasing the intermolecular electrostatic repulsion and ultimately facilitating the gradual formation of a gel network as intermolecular hydrogen bonding forms and stabilizes. Considering that the T_melt_ of AP gels is almost independent of pH variation and comparable to that of gelatin, AP is anticipated to exhibit great potential in the food industry as a substitute for gelatin. However, to fully realize this application potential, further investigations into several critical practical parameters are imperative, encompassing sensory testing, in vitro/in vivo digestibility assays, allergenicity evaluations, and regulatory compliance assessments across major food markets.

## Data Availability

The original contributions presented in the study are included in the article/Supplementary Material, further inquiries can be directed to the corresponding author.

## Supplementary Materials

The following supporting information can be downloaded at: https://www.mdpi.com/article/10.3390/foods15061076/s1, Figure S1: Appearance of AP solutions under different pH values at 25 °C and 4 °C; Figure S2: Zeta-potential of AP (0.1%, *w/v*) with different amounts of CaCl_2_ additions at 4 °C. For statistical validity, all measurements were replicated nine times (n = 3) independently.

## Abbreviations

The following abbreviations are used in this manuscript:

AP	Apple polysaccharide
GalA	Glucuronic acid content
DM	Degree of methoxylation
Mw	Molecular weight
SEM	Scanning electron microscope
TG	Thermogravimetry
DTG	Derivative thermogravimetry
FTIR	Fourier transform infrared spectroscopy
XRD	X-ray diffraction
AFM	Atomic force microscope
ITC	Isothermal titration calorimetry
EPR	Electron paramagnetic resonance
ESR	Electron spin resonance
LMP	Low methoxyl pectin
