# Supplementary material for: The Gelation Mechanism of an Apple Polysaccharide at Ambient Temperature as Induced by Ca2+
Source: Foods. 2026 Mar 19;15(6):1076. doi: 10.3390/foods15061076 (PMC13025593; doi:10.3390/foods15061076)
Supplement: Supplementary file 1 [file foods-15-01076-s001.zip › Supplementary Material.pdf]

Supplementary materials for:

The Gelation Mechanism of an Apple Polysaccharide at Ambient Temperature as Induced by Ca2+

Shuai Luo [1], Junhao Qiu [2,3], Shuaida Wang [1], Xi Yang [2,3] and Haopeng Wang [2,3,*]

1 College of Food Engineering and Nutritional Science, Shaanxi Normal University, 620 West Chang'an Avenue, Xi'an 710119, China; luoshuai@snnu.edu.cn (S.L.); sdwang618@snnu.edu.cn (S.W.)

2 State Key Laboratory for Quality and Safety of Agro-Products, Ningbo 315800, China; junhqiu@163.com (J.Q.); yangxi1@nbu.edu.cn (X.Y.)

3 Zhejiang-Malaysia Joint Research Laboratory for Agricultural Product Processing and Nutrition, College of Food Science and Engineering, Ningbo University, Ningbo 315800, China

* Correspondence: wanghaopeng@nbu.edu.cn

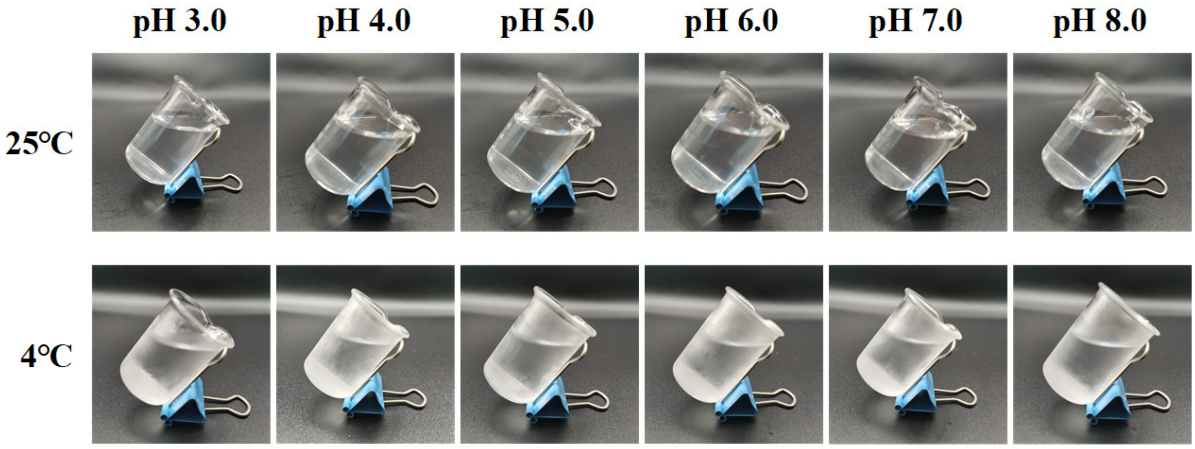

Figure S1. Appearance of AP solutions under different pH values at 25℃ and 4℃.

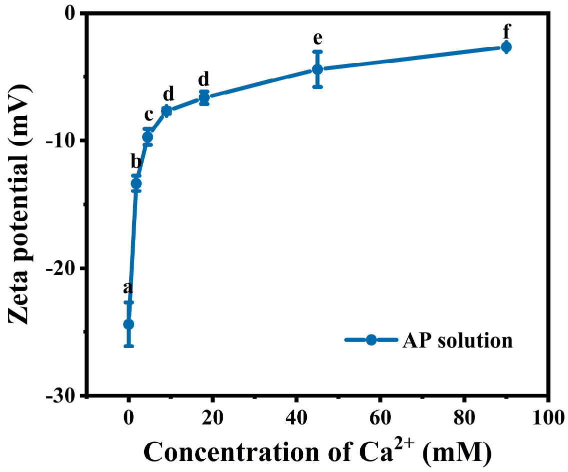

Figure S2. Zeta potential of AP (0.1%, w/v) with different amount of $CaCl_2$ additions at 4℃. For statistical validity, all measurements were replicated nine times (n = 3) independently.
